# Repairing the medial meniscus posterior root during open-wedge high tibial osteotomy does not significantly affect clinical outcomes

**DOI:** 10.3389/fsurg.2025.1672154

**Published:** 2025-10-02

**Authors:** Conglei Dong, Lingce Kong, Yingzhen Niu, Jiangtao Dong

**Affiliations:** Department of Orthopaedic Surgery, Third Hospital of Hebei Medical University, Shijiazhuang, Hebei, China

**Keywords:** medial meniscus posterior root tear, open-wedge high tibial osteotomy, cartilage damage, limb alignment, clinical efficacy

## Abstract

**Objectives:**

This study aimed to evaluate the effectiveness of medial meniscus posterior root tear (MMPRT) repair during open-wedge high tibial osteotomy (OWHTO) by investigating MMPRT healing and clinical outcomes. It also aimed to explore the impact of lower limb alignment correction on MMPRT healing in unrepaired cases.

**Methods:**

A total of 157 patients (68 males and 89 females) were included, with an average age of 57.0 ± 6.66 years and an average postoperative follow-up duration of 22.1 ± 2.92 months, who underwent OWHTO followed by second-look arthroscopy. Patients were divided into two groups: the OWHTO with MMPRT repair group (*n* = 82) and the OWHTO-only group (*n* = 75). Each group was further divided into Fujisawa subgroup and neutral subgroups to assess the healing of MMPRT and clinical outcomes.

**Results:**

The overall MMPRT healing outcomes in the OWHTO with MMPRT repair group were similar to the OWHTO-only group. Cartilage damage showed no intergroup differences. Functional improvements were equivalent between groups. Subgroup analyses revealed differential outcomes: Fujisawa subgroup exhibits superior healing in isolated OWHTO, but not in combined procedures.

**Conclusion:**

Mid-term clinical outcomes were comparable between OWHTO combined with MMPRT pull-out repair and isolated OWHTO. For patients undergoing isolated OWHTO, mechanical axis correction targeting the Fujisawa point is significantly more conducive to MMPRT healing than neutral alignment. Consider prioritizing MMPRT repair for young patients or those with high activity demands. When MMPRT repair is not performed, it is recommended to target the correction of knee alignment to the Fujisawa point.

## Introduction

Medial meniscus posterior root tear (MMPRT) is a common knee joint injury (1), often causes loss of medial meniscus hoop tension, increasing medial compartment contact stress and accelerating osteoarthritis (2), making its surgical repair a focus in sports medicine.

Open-wedge high tibial osteotomy (OWHTO) corrects varus alignment and reduces medial compartment pressure to slow osteoarthritis. However, there is no consensus on whether to repair MMPRT during OWHTO, as the value of combined repair remains unclear. Nha et al. (3) reported that partial healing of MMPRT could be achieved through alignment correction alone, without specific intervention for the meniscus. This finding suggests that the necessity for MMPRT repair during OWHTO may not be as critical as previously assumed. Currently, two key knowledge gaps exist: (1) Whether MMPRT repair during OWHTO provides additional benefits for meniscal healing compared to OWHTO-only; (2) How lower limb alignment correction affects MMPRT natural healing in unrepaired patients.

This study aimed to evaluate the efficacy of concomitant MMPRT repair during OWHTO through second-look arthroscopy and to investigate the impact of lower limb alignment correction on MMPRT healing and cartilage damage. We hypothesized that there would be no significant difference in MMPRT healing outcomes between OWHTO with concomitant MMPRT repair and OWHTO alone. Furthermore, we postulated that when MMPRT is left unrepaired, mechanical axis correction to the Fujisawa point would yield superior clinical outcomes compared with neutral alignment correction. The innovation of this study is the use of second-look arthroscopy to directly assess meniscal healing and subgroup analysis based on postoperative alignment to explore alignment-healing associations.

## Materials and methods

### Patient inclusion

This retrospective study was approved by the Ethics Committee of our hospital (Ke2024-092-1) and analyzed the medical records of patients diagnosed with knee osteoarthritis (KOA) and symptomatic MMPRTs who underwent OWHTO between January 2023 and December 2023. No second-look arthroscopies were performed solely for research purposes; all such procedures were conducted concurrently with clinically indicated hardware removal to avoid exposing patients to unnecessary surgical risk. Notably, this study incorporates prospective elements within a retrospective framework (e.g., standardized surgical protocol, uniform 1/3/6/12-month follow-up schedule, mandatory second-look arthroscopy during hardware removal), as these procedures were pre-defined and consistently implemented before data collection.

The inclusion criteria for the study were as follows: (1) Patients diagnosed with medial compartment osteoarthritis accompanied by symptomatic MMPRTs. (2) Varus malalignment with a varus deformity <15°. (3) MMPRTs confirmed through preoperative magnetic resonance imaging (MRI) and intraoperative arthroscopy. (4) Radiographic Kellgren-Lawrence (K-L) grade < IV. (5) Near-normal joint range of motion (flexion contracture <10°).

The exclusion criteria for the study were as follows: (1) Patients with severe medial compartment KOA and complete loss of the medial joint space (K-L grade IV). (2) Patients with lateral compartment osteoarthritis or patellofemoral osteoarthritis. (3) Obese patients with a body mass index (BMI) > 30. (4) Patients with ligament injuries or knee joint instability. (5) Patients with knee varus deformity >15° or flexion contracture > 10°. (6) Patients with a history of prior knee surgery. (7) Patients who did not undergo second-look arthroscopy. (8) Postoperative lower limb alignment deviated from both the 50%–55% and 60%–65% ranges of tibial plateau width.

**Figure 1:**
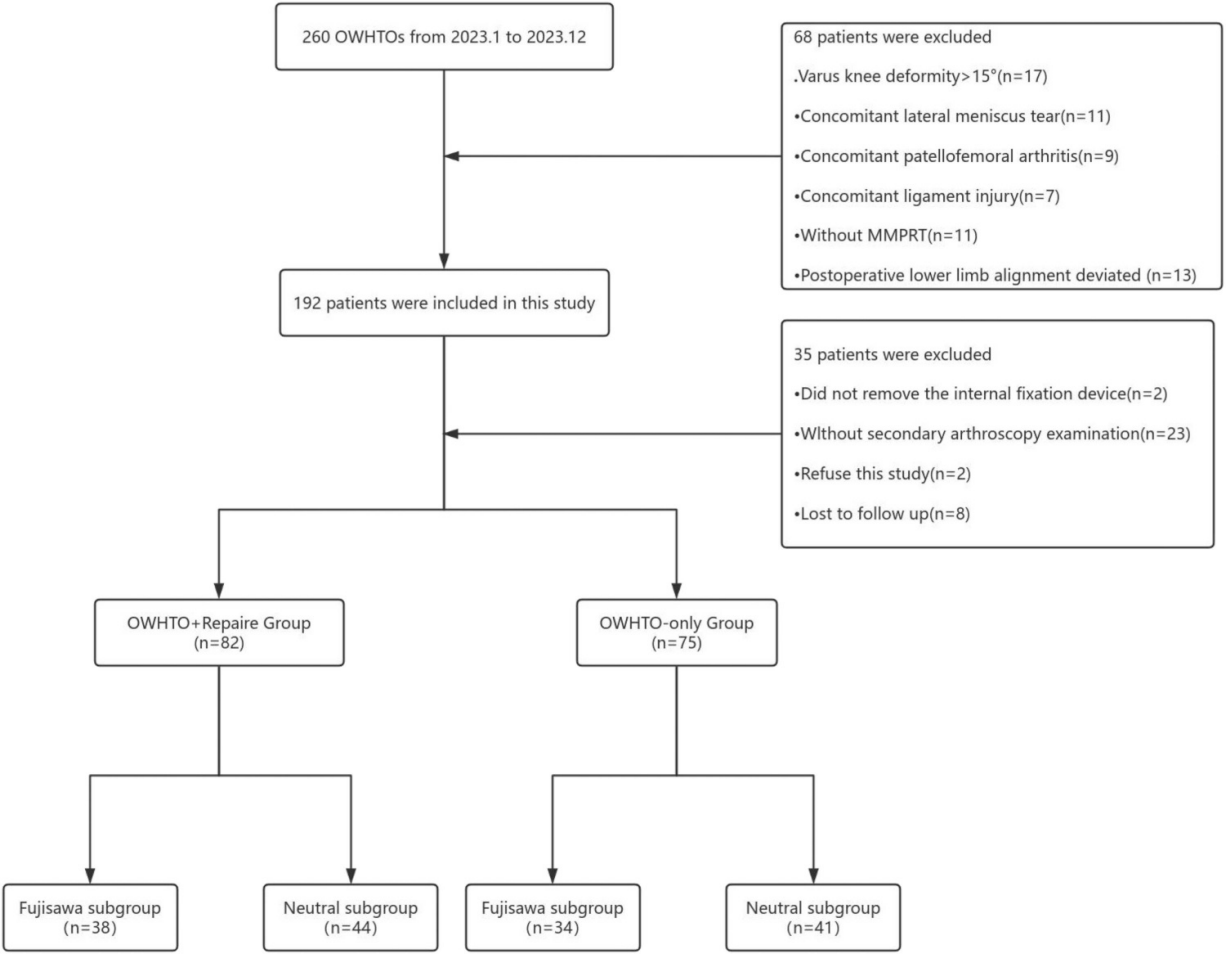
Patients inclusion flowchart. OWHTO, open wedge high tibial Osteotomy; MMPRT, medial meniscus posterior root tear.

**Figure 2:**
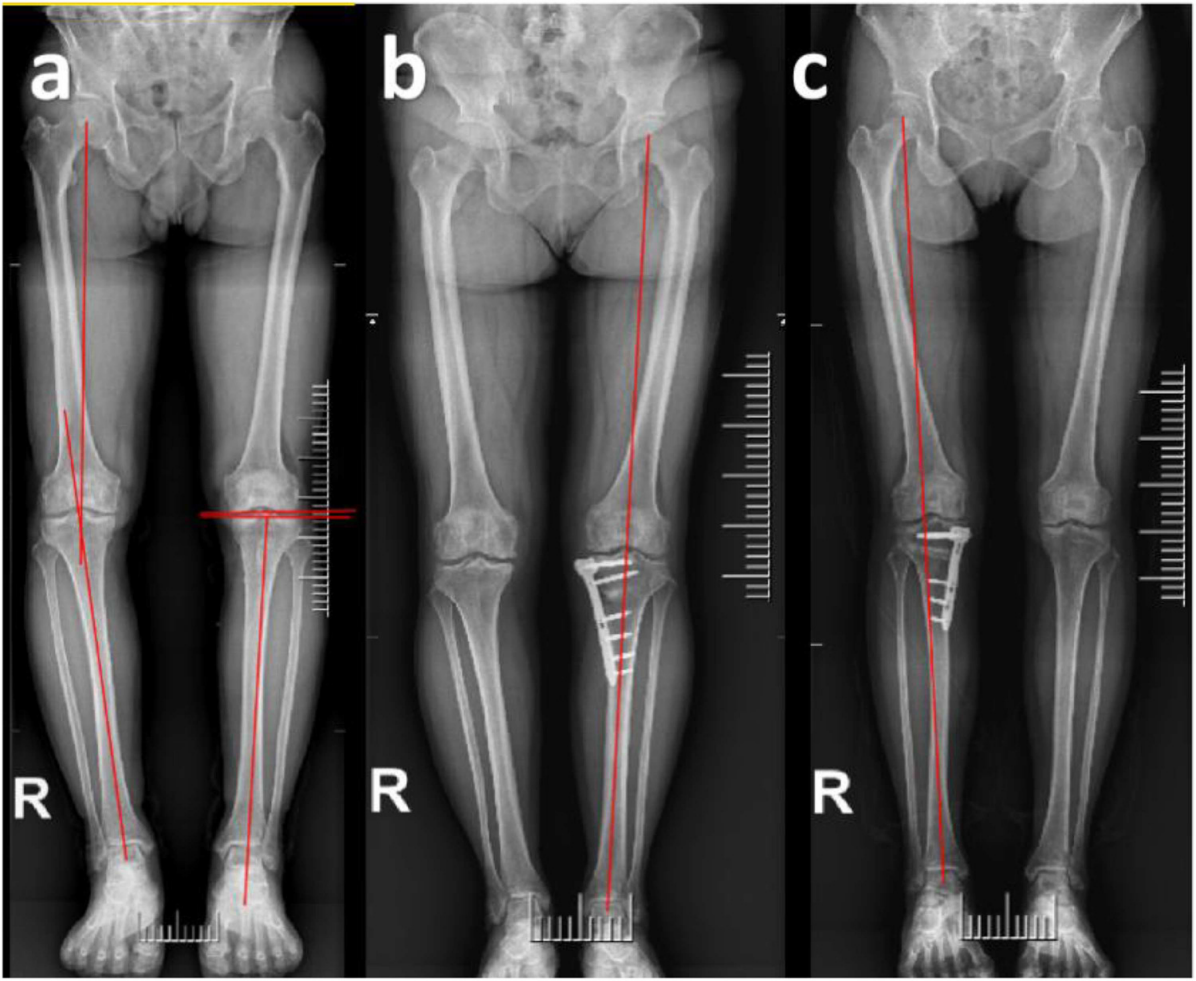
Radiographic evaluation examples. **(a)** Schematic diagram for measuring HKA, MPTA and JLCA. The hip-knee-ankle (HKA) angle was defined as the angle between the line from the center of the femoral head to the midpoint between the tibia intercondylar eminences and the line from the midpoint between the tibia intercondylar eminences center to the talus joint surface center. The medial proximal tibial angle (MPTA) was defined as the angle between the mechanical axis of the tibia and the proximal articular surface of the tibia. The joint line convergence angle (JLCA) is defined as the angle formed between the distal femoral articular surface and the proximal tibial articular surface in the coronal plane. **(b,c)** Postoperative lower limb alignment passing through 50%–55% of the tibial plateau width was classified into the Neutral subgroup, while alignment passing through 60%–65% of the tibial plateau width was assigned to the Fujisawa subgroup.

A total of 157 patients who underwent open-wedge high tibial osteotomy (OWHTO) followed by second-look arthroscopy were included as the study cohort. The patients were divided into two groups: the OWHTO with MMPRT repair group (*n* = 82) and the OWHTO-only group (*n* = 75). Patients who underwent MMPRT repair using the pull-out technique during OWHTO were assigned to the repair group, while those who only received debridement of the degenerative portion of MMPRT without repair were assigned to the non-repair group. Whether to repair the meniscus is determined based on preoperative imaging observations, intraoperative meniscus tear conditions, and patient preferences. All results were obtained retrospectively from medical records. Additionally, each group was further subdivided into Fujisawa and Neutral subgroups. The patient selection process is illustrated in Figure 1. Postoperative lower limb alignment passing through 60%–65% of the tibial plateau width was classified into the Fujisawa subgroup, while alignment passing through 50%–55% of the tibial plateau width was assigned to the neutral subgroup (4, 5) (Figure 2). The alignment results were obtained from postoperative measurements. Cases where the postoperative alignment did not meet the standards were excluded, and this information is presented in Figure 1.

### Surgical technique and postoperative rehabilitation

All surgeries (OWHTO) and second-look arthroscopies were performed by the same experienced knee surgeon. No additional surgical procedures, such as microfracture, chondroplasty, or autologous osteochondral transplantation, were performed apart from MMPRT repair. Furthermore, no bone grafts were implanted in the osteotomy gap.

Following the routine diagnostic arthroscopy, a scaled probe was used during both OWHTO and the second-look arthroscopy to measure the size of cartilage damage.

A longitudinal incision of approximately 7 cm was made on the anteromedial proximal tibia. After the complete release of the superficial attachment of the pes anserinus at the medial collateral ligament insertion, a Hoffmann retractor was placed to protect the neurovascular structures posterior to the osteotomy line. The first oblique osteotomy was performed approximately 35 mm from the medial tibial plateau, directed toward the fibular head, stopping 5 mm from the lateral cortical margin of the proximal tibia. A second osteotomy (biplane osteotomy) was performed at an angle of 110° relative to the first osteotomy line at the patellar tendon insertion, in the coronal plane. The osteotomy site was gradually opened at an appropriate angle, followed by fixed using a locking plate-screw system.

Subsequently, MMPRT repair was performed using the pull-out technique. The torn edges of the meniscus were debrided with a vertical mattress technique and sutured using Ethibond No. 2 sutures. A guidewire was passed through the anterolateral cortex of the proximal tibia to the inferior surface of the posterior medial meniscus root footprint to create a tibial tunnel. The tunnel was then reamed (4.5 mm) over the guidewire. The Ethibond suture was pulled through the tibial tunnel, and the stability of the meniscus root was reassessed. The suture was tensioned under adequate traction and fixed to the anterolateral tibial cortex using a suspensory fixation. In the isolated OWHTO group, the MMPRT was simply debrided to freshen the degenerative portion of the meniscal tear.

For the OWHTO with MMPRT repair group, weight-bearing was delayed until 4 weeks postoperatively, at this point partial weight-bearing with crutches was allowed. Full weight-bearing was permitted starting at 8 weeks postoperatively. For the OWHTO-only group, immediate partial weight-bearing with crutches was encouraged postoperatively. If the patient could bear full weight, they were permitted to do so.

Knee flexion exercises were allowed with the use of a knee brace to limit excessive movement. All patients were instructed to maintain knee flexion at 30° for 1 week and at 90° for 4 weeks. The knee brace was removed at 4 weeks postoperatively. Immediately after surgery, all patients began quadriceps strengthening exercises to prevent muscle atrophy. Both groups were instructed to avoid squatting for 3 months postoperatively, and they were allowed to return to sports activities after 6 months.

### Clinical and radiological evaluation

Patients were followed up at 1, 3, 6, and 12 months postoperatively, and subsequently every 6 months, with weight-bearing compliance assessed via gait observation and patient self-reports; adherence rate was >90% in both groups. Preoperative and at the last follow-up, knee flexion contracture and range of motion were measured using a long-arm goniometer. Knee function was assessed using the Lysholm score and the Tegner score at both preoperative and last follow-up visits. Knee pain was evaluated using the Visual Analog Scale (VAS). For these scores, the recommended Minimum Clinically Important Difference (MCID) values are +25.4 for the Lysholm score and −2.46 for the VAS pain score. Any improvement exceeding these values indicates clinical significance (6). All clinical assessments were performed by two experienced, non-surgical observers and conducted in accordance with the blinding method. Radiological and clinical evaluations were performed immediately before OWHTO and second-look arthroscopy. The hip-knee-ankle (HKA) angle was defined as the angle between the line from the center of the femoral head to the midpoint between the tibia intercondylar eminences and the line from the midpoint between the tibia intercondylar eminences center to the talus joint surface center (7). The joint line convergence angle (JLCA) is defined as the angle formed between the distal femoral articular surface and the proximal tibial articular surface in the coronal plane (8). The medial proximal tibial angle (MPTA) was defined as the angle between the mechanical axis of the tibia and the proximal articular surface of the tibia in the coronal plane (9) (Figure 2). The radiological severity of osteoarthritis was assessed using the Kellgren-Lawrence (K-L) grading system (10).

These measurements were taken on full-length standing radiographs of the lower limb. All radiological measurements were performed by two experienced orthopedic surgeons using RadiAnt DICOM Viewer software (Medixant Ltd.) both preoperatively and postoperatively. Patient information was recorded, and the angular precision was 0.1°. Six weeks after the initial measurement, repeated measurements were conducted by the same authors to calculate the intraobserver reliability of the intraclass correlation coeffcient (ICC). A value of ICC ≥ 0.8 was considered good and ≥ 0.9 excellent.

### Second-look arthroscopy evaluation

**Figure 3:**
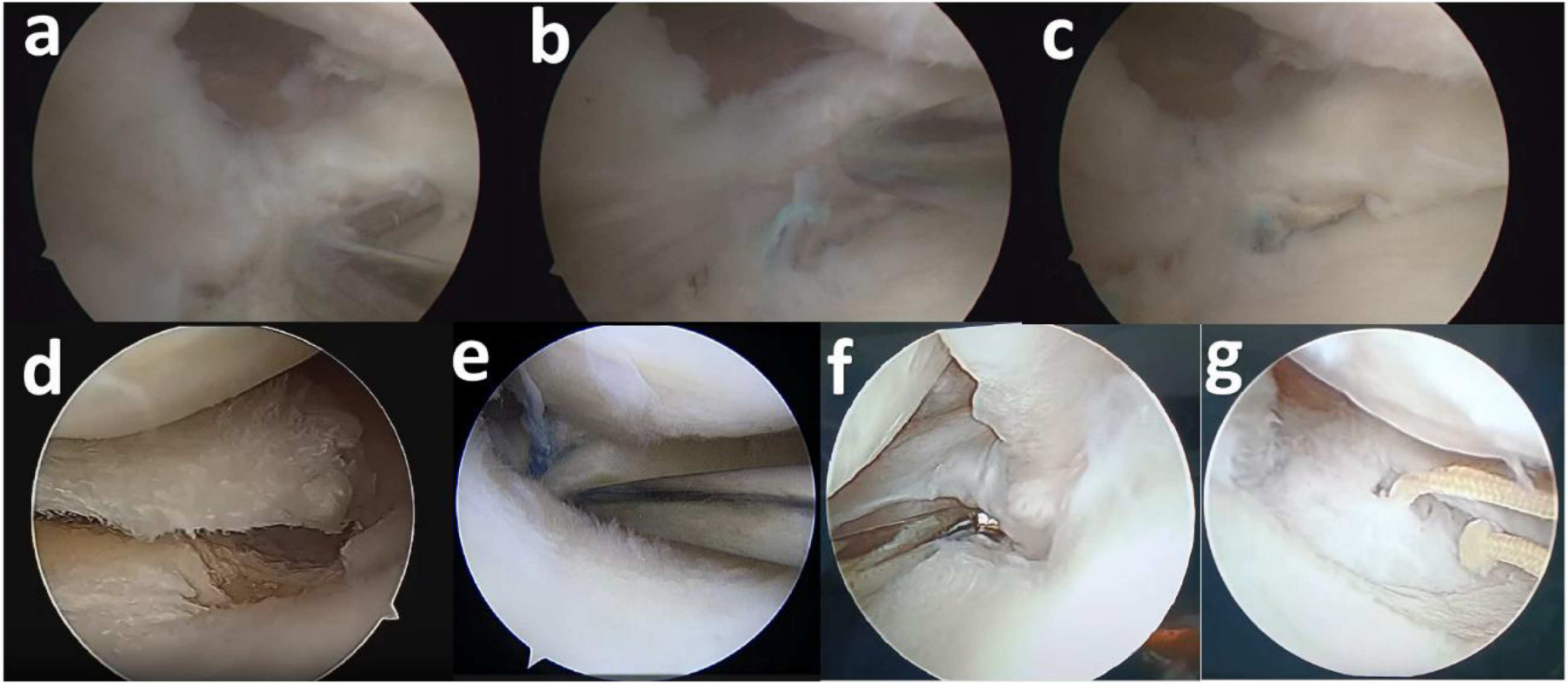
Example of arthroscopic examination. **(a)** Anterior-posterior width of the repaired posterior root. **(b)** Stability of the repaired posterior root. **(c)** Synovial coverage of the posterior root. **(d)** Initial root tears. **(e)** Pull-out repair **(f)** Healed root **(g)** Nonhealed root.

All patients underwent second-look arthroscopy at least 1 year postoperatively, during which metal fixation devices were removed. The degree of cartilage degeneration in the medial compartment was systematically documented using the Outerbridge classification during both arthroscopic procedures. Scoring was also performed for posterior root width, stability and the degree of synovial coverage at the root attachment site. The healing of the meniscus after MMPRT repair was evaluated using the semi-quantitative arthroscopic scoring system established by Furumatsu et al. (11). The scale consists of three evaluation criteria: (1) The anterior-posterior width of the posterior root after healing: classified as broad (>5 mm, 4 points), narrow (2–5 mm, 2 points), or filamentous (<2 mm, 0 points). (2) Stability of the posterior root after healing: evaluated as good (no posterior root lift at 20° knee flexion, 4 points), acceptable (no posterior root lift at 60° knee flexion, 3 points), loose (no anterior drawing at 20° knee flexion, 2 points), useless (acceptable continuity of the posterior root, 1 point), or completely unstable (unacceptable continuity of the posterior root, 0 points). (3) Synovial coverage of the posterior root: classified as good (complete synovial coverage, 2 points), fair (slight synovial coverage, 1 point), or poor (almost no synovial coverage, 0 points) (Figure 3). In addition to the operating surgeon, another fixed physician also evaluated the healing status of MMPRT to assess the inter-observer reliability.

### Statistical analysis

All statistical analyses were performed using SPSS Statistics 26 (IBM Corp). The normality of distribution and homogeneity of variance for all variables were tested using the Kolmogorov–Smirnov test. Independent sample t-tests were used to compare clinical outcomes and radiological parameters between groups. Paired t-tests were used to compare preoperative and final follow-up clinical outcomes and radiological measurements. The chi-square test was used to examine categorical variables, such as K-L grades and Outerbridge grades, between groups. When significant differences were found using the Kruskal–Wallis test, comparisons between groups were performed using the Mann–Whitney *U* test. *P* < 0.05 was considered significant. Based on the difference in the posterior root stability score, a type I error rate of 5% and a type II error rate of 20% (80% power), the sample size of 32 patients per group was calculated.

## Results

Between January 2023 and December 2023, a total of 260 patients underwent OWHTO at our institution. Based on predefined exclusion criteria, 68 patients were excluded from the study cohort for the following reasons: varus deformity exceeding 10° (*n* = 17), concomitant lateral meniscus injury (*n* = 11), coexisting patellofemoral osteoarthritis (*n* = 9), anterior cruciate ligament injury (*n* = 7), postoperative lower limb alignment deviated (*n* = 13) and absence of MMPRT (*n* = 11). Furthermore, an additional 35 patients were excluded due to: failure to return for metal fixation devices removed (*n* = 2), absence of second-look arthroscopy (*n* = 23), refusal to participate in the study (*n* = 2), and loss to follow-up (*n* = 8). Finally, a total of 157 patients (68 males and 89 females) were included, with an average age of 57.0 ± 6.66 years and an average postoperative follow-up duration of 22.1 ± 2.92 months. Demographic data of the OWHTO with MMPRT repair group and the OWHTO-only group were compared (Table 1). The intrarater and interrater reliability for the imaging measurements and arthroscopic assessments had an ICC of >0.9 (range 0.907–0.964), indicating high reliability of the radiological measurements.

**Table 1:** General characteristics of patients in the final cohort (*n* = 157).

Characteristics	OWHTO + Repair (*n* = 82)	OWHTO-only (*n* = 75)	*P*
Gender (Male/Female)	36/46	32/43	0.876
Age (year)	57.4 ± 7.04	56.6 ± 6.23	0.443
BMI (weight/height squared)	25.7 ± 2.48	26.5 ± 2.38	0.063
Side (Left/Right)	38/44	38/37	0.588
Follow-up Time (month)	22.0 ± 2.97	22.1 ± 2.90	0.949
K-L Grading (1/2/3)	12/29/41	11/23/41	0.809
Flexion Contracture(°)			
Preoperative	3.3 ± 0.98	3.2 ± 0.89	0.841
Postoperative	2.2 ± 1.12	2.2 ± 1.11	0.966
*P*	**<0.01**	**<0.01**	
Range of Motion (°)			
Preoperative	128.2 ± 3.81	127.8 ± 3.99	0.558
Postoperative	131.8 ± 4.01	132.0 ± 3.69	0.777
*P*	**<0.01**	**<0.01**	

Bold represents statistically significant differences.

Preoperatively, the Lysholm scores for the OWHTO with MMPRT repair group and the OWHTO-only group were 54.1 ± 9.59 and 55.6 ± 9.75, respectively. The Tegner scores were 1.1 ± 1.06 and 1.2 ± 1.15, and the VAS scores were 8.0 ± 1.08 and 7.9 ± 1.13. There were no significant differences between the two groups in terms of subjective sensations and pain prior to surgery (*P* > 0.05).

At the final follow-up, the Lysholm scores for the two groups were 87.7 ± 6.67 and 86.4 ± 6.89, respectively, the Tegner scores were 3.7 ± 0.89 and 3.8 ± 0.87, and the VAS scores were 1.4 ± 0.85 and 1.5 ± 0.81. Both groups showed significant improvements compared to preoperative scores, but no significant differences were observed between the two groups (*P* > 0.05). In the mid-term, there was no significant improvement in clinical scores with the use of MMPRT repair in OWHTO compared to OWHTO alone (Table 2).

**Table 2:** Comparison of treatment efficacy between OWHTO with MMPRT repair group and OWHTO-only group.

Outcome measures	OWHTO + Repair (*n* = 82)	OWHTO-only (*n* = 75)	*P*	Effect Sizes (CI)
HKA (°)				
Preoperative	173.2 ± 2.35	173.1 ± 2.35	0.906	−0.04 (−0.34, 0.26)
Postoperative	183.2 ± 2.14	183.1 ± 1.92	0.999	0.05 (−0.25, 0.35)
*P*	**<0.001**	**<0.001**		
MPTA (°)				
Preoperative	83.3 ± 1.38	83.2 ± 1.46	0.751	0.07 (−0.23, 0.37)
Postoperative	89.1 ± 2.38	89.4 ± 2.33	0.453	−0.13 (−0.43, 0.17)
*P*	**<0.001**	**<0.001**		
JLCA (°)				
Preoperative	**3.2** ± **0.91**	**3.4** ± **0.80**	0.161	−0.23(−0.53, 0.07)
Postoperative	**2.5** ± **0.87**	**2.3** ± **0.83**	0.314	0.24 (−0.06, 0.54)
*P*	**<0.001**	**<0.001**		
Lysholm Score				
Preoperative	54.1 ± 9.59	55.6 ± 9.75	0.316	−0.16 (−0.46, 0.14)
Postoperative	87.7 ± 6.67	86.4 ± 6.89	0.232	0.19 (−0.11, 0.49)
*P*	**<0.001**	**<0.001**		
Tegner Score				
Preoperative	1.1 ± 1.06	1.2 ± 1.15	0.385	−0.09 (−0.39, 0.21)
Postoperative	3.7 ± 0.89	3.8 ± 0.87	0.526	−0.11 (−0.41, 0.19)
*P*	**<0.001**	**<0.001**		
VAS Score				
Preoperative	8.0 ± 1.08	7.9 ± 1.13	0.562	0.09 (−0.21, 0.39)
Postoperative	1.4 ± 0.85	1.5 ± 0.81	0.683	−0.12 (−0.42, 0.18)
*P*	**<0.001**	**<0.001**		
Outerbridge Grade (1/2/3/4)				
Preoperative	9/27/38/8	9/23/33/10	0.900	50.8% (43.2%, 58.4%)
Postoperative	11/28/37/6	7/27/33/8	0.774	51.5% (43.9%, 59.1%)
*P*	0.896	0.813		
Posterior Root Width	3.05 ± 1.23	2.78 ± 1.23	0.162	0.22 (−0.08, 0.52)
Posterior Root Stability	2.99 ± 1.08	2.75 ± 1.24	0.196	0.21 (−0.09, 0.51)
Synovial Coverage	1.49 ± 0.63	1.52 ± 0.62	0.749	−0.05 (−0.35, 0.25)

Bold represents statistically significant differences.

There was no significant difference in the degree of medial meniscus posterior root healing between the OWHTO with MMPRT repair group and the OWHTO-only group (*P* > 0.05). This result suggests that adding MMPRT repair to OWHTO did not significantly improve healing outcomes (Table 2). In the subsequent subgroup analysis, when MMPRT repair was performed, there was no difference in MMPRT healing between the Fujisawa subgroup (*n* = 38) and the Neutral subgroup (*n* = 44) (*P* > 0.05). However, for patients who did not undergo MMPRT repair, the healing degree in the Neutral subgroup (*n* = 41) was worse than that in the Fujisawa subgroup (*n* = 34), with a statistically significant difference (*P* = 0.026 and 0.039). The findings indicate that in the absence of MMPRT repair, the degree of correction significantly influences the healing outcome. A mild valgus alignment achieved by correcting the lower limb mechanical axis to the vicinity of the Fujisawa point was demonstrated to be more conducive to healing. Additionally, there was no significant difference in cartilage damage between the OWHTO with MMPRT repair group and the OWHTO-only group (*P* > 0.05). This indicates that in the current study, MMPRT repair did not significantly improve cartilage damage (Table 3).

**Table 3:** Comparison of clinical efficacy between Fujisawa subgroup and neutral subgroup.

Outcome measures	OWHTO + Repair (*n* = 82)		*P*	Effect Sizes (CI)
	Fujisawa subgroup (*n* = 38)	Neutral subgroup (*n* = 44)		
Outerbridge Grade (1/2/3/4)	5/13/17/3	6/15/20/3	0.998	50.2% (40.1%, 60.3%)
Posterior Root Width	3.11 ± 1.29	3.00 ± 1.18	0.701	0.09 (−0.32, 0.50)
Posterior Root Stability	2.92 ± 1.02	3.05 ± 1.14	0.607	−0.12 (−0.43, 0.19)
Synovial Coverage	1.50 ± 0.65	1.48 ± 0.63	0.872	0.03 (−0.38, 0.44)
	OWHTO-only (*n* = 75)			
	Fujisawa subgroup (*n* = 34)	Neutral subgroup (*n* = 41)		
Outerbridge Grade (1/2/3/4)	3/13/15/3	4/14/18/5	0.960	51.1% (40.8%, 61.4%)
Posterior Root Width	3.12 ± 1.01	2.49 ± 1.33	**0**.**026**	0.52 (0.08, 0.96)
Posterior Root Stability	3.06 ± 0.92	2.49 ± 1.42	**0**.**039**	0.48 (0.04, 0.92)
Synovial Coverage	1.53 ± 0.61	1.51 ± 0.64	0.906	0.03 (−0.38, 0.44)

Bold represents statistically significant differences.

## Discussion

In this study, Repairing for MMPRTs during OWHTO did not demonstrate significant clinical benefits. However, the key novel finding of this study is that in patients undergoing isolated OWHTO, mechanical axis correction to the Fujisawa point significantly improves MMPRT healing compared to neutral alignment. This finding is more meaningful than the negative result, as it provides a clear, actionable alignment target for managing unrepaired MMPRT during OWHTO. The primary strength of this study lies in the utilization of secondary arthroscopy during metal fixation device removal to assess meniscal healing and cartilage damage, which demonstrates superior accuracy compared to imaging evaluation alone.

The meniscal repair rates for MMPRT vary widely across studies (12, 13). Many previous studies did not simultaneously consider the effects of lower limb alignment and repair, making it difficult to determine whether high healing rates were due to meniscal repair or OWHTO-induced alignment correction (14).

Finite element biomechanical studies have shown that MMPRT repair significantly reduces peak contact pressure in the medial compartment (15). Similarly, cadaveric biomechanical analyses have demonstrated that MMPRT repair increases tibiofemoral contact area (16). Research by Ke et al. (17) and Lee et al. (18) suggested that OWHTO combined with MMPRT repair achieved higher meniscal healing rates, which contrasts with our findings. However, their evaluation of posterior root healing was based on the qualitative assessment by Seo et al. (19), whereas we adopted a semi-quantitative scoring system for comparison, which might lead to divergent conclusions. Guo et al. (20) demonstrated that OWHTO combined with MMPRT repair led to better recovery of athletic abilities in younger patients. However, in our cohort, the majority of OWHTO surgeries were performed as part of stepwise treatment for osteoarthritis after conservative therapy had failed, with most patients being older than 55, and many over 65. Patients in this age group typically present with early to moderate-stage osteoarthritis of the medial compartment, frequently accompanied by MMPRT. The majority of these patients are suitable for OWHTO, which serves as an effective joint-preserving intervention to delay or potentially avoid the need for TKA. This patient population represent a suitable target group for OWHTO. This surgical approach appears to be more appropriate for younger, more physically active patients who typically demonstrate better healing potential and functional recovery (21). Furthermore, considering the relatively higher economic costs associated with MMPRT repair, patient selection should prioritize individuals who are most likely to derive significant clinical benefits from the combined procedure.

Meanwhile, Lee et al. (18) found that MMPRT repair did not significantly affect functional scores during postoperative follow-up, which is consistent with our observations. And they demonstrated that the repair of MMPRTs did not significantly improve medial meniscal extrusion. A meta-analysis on MMPRT repair reported significant improvements in postoperative subjective clinical scores compared to preoperative states (22). However, it also highlighted that MMPRT repair did not effectively reduce meniscal deformation compared to meniscectomy. In our study, the presence or absence of MMPRT repair did not significantly affect the healing degree. The Lysholm score was the most frequently reported clinical outcome measure, and our postoperative scores were consistent with previously published results (23). In our study, no significant differences were observed in functional outcomes between groups, with both demonstrating substantial improvement compared to preoperative baselines. These findings suggest that the addition of MMPRT repair to OWHTO is not significantly associated with improvements in postoperative knee function. Therefore, we believe that MMPRT repair should be prioritized for young patients with high activity demands and intact meniscal tissue. MMPRT repair during OWHTO may still be considered to maximize long-term functional recovery and reduce the risk of early meniscal re-tear. For elderly patients with degenerative MMPRT limiting repair tolerance, surgeons should prioritize OWHTO with alignment correction to the Fujisawa point—this approach avoids unnecessary surgical time, cost, and rehabilitation delays associated with repair, while still optimizing MMPRT healing through mechanical axis adjustment.

Notably, among patients subjected to isolated OWHTO, the Fujisawa subgroup demonstrated superior healing outcomes compared to the Neutral subgroup. The mechanical axis with mild valgus alignment facilitated more effective load transfer to the lateral compartment, thereby reducing localized microstrain on the medial meniscus and creating a favorable biomechanical environment for MMPRT healing (24, 25). Furthermore, lateral shift of the lower limb mechanical axis may results in widening of the medial joint space, which enhances synovial fluid diffusion and reduces local concentrations of inflammatory mediators, and creates a protective environment that effectively mitigates meniscal degeneration (26, 27). Although MMPRT leads to loss of meniscal hoop tension and reduced tibiofemoral contact area, the varus-aligned HKA and increased JLCA play a more prominent role in joint degeneration, resulting in elevated pressure within the affected medial tibiofemoral compartment (28). However, only the OWHTO-only group benefited from the Fujisawa point. We believe this is because the repaired MMPRT regains circumferential tension through surgical fixation, reducing the dependence of healing on alignment. In contrast, the unrepaired MMPRT relies entirely on alignment at the Fujisawa point to reduce medial compartment pressure and promote natural healing, resulting in a more significant impact. We propose that proper lower limb alignment correction and the release of medial compartment pressure are key factors for MMPRT healing. When MMPRT is not repaired, surgeons should target alignment at the Fujisawa point to optimize natural healing and avoid neutral alignment. It is worth noting that the range of correction needs to be precisely controlled to avoid lateral compartment overload and patellofemoral complications (29, 30). For patients undergoing neutral alignment correction, secure fixation and repair of the posterior root of the meniscus may play a critical role in achieving better healing outcomes.

The restoration of meniscal hoop tension depends on the actual healing of the reduced position. If meniscal extrusion persists, it is unlikely to restore hoop tension or improve tibiofemoral contact area (31). Although previous studies suggested that MMPRT repair might promote cartilage regeneration, our findings indicate that the degree of cartilage damage is independent of posterior root repair. Instead, the pressure reduction achieved through OWHTO alone can lead to comparable levels of cartilage healing. Therefore, we propose that lower limb alignment correction holds more profound clinical significance than MMPRT repair for this elderly patient population.

This study has several limitations. First, the cohort size was relatively small, and the study was retrospective with an average follow-up duration of 22.1 ± 2.92 months—this is relatively short, as osteoarthritis studies typically require ≥5 years of follow-up to assess cartilage degeneration progression. Long-term outcomes need to be verified by extended follow-up. Notably, the strict exclusion criteria (postoperative misalignment, *n* = 13; no second-look arthroscopy, *n* = 23) resulted in a highly selected cohort: this sample likely overrepresents patients with good surgical compliance, favorable baseline joint conditions, and access to follow-up care, which may limit the generalizability of our findings to broader clinical populations. Excluding these patients also increases the risk of potential Type II error. Second, although the arthroscopic classification system employs a semi-quantitative scoring method, it may still be influenced by the surgeon's subjective judgment. However, intraoperative findings were recorded on video and reviewed by multiple experienced orthopedic surgeons to minimize observational bias. Third, this study exclusively employed the pull-out technique for MMPRT repair without evaluating the impact of alternative suture methods and subgroup classification was based on postoperative mechanical axis alignment near or at the Fujisawa point vs. neutral position. Variations in correction targets adopted by different studies may contribute to outcome heterogeneity. Notably, we did not perform formal adjustment for multiple comparisons for parallel inter-group and subgroup analyses. Thus, despite the use of an independent samples *t*-test, the risk of type I error may be inflated. Fourth, The repair group adopted a delayed weight-bearing protocol, while the non-repair group implemented immediate partial weight-bearing postoperatively (32). Although the safety of both protocols has been confirmed through long-term follow-up in clinical practice and neither exerts an independent impact on healing outcomes, they may indeed act as confounding factors in statistics. This inability to adjust for potential confounding factors (e.g., rehabilitation adherence) remains a key limitation that may affect the robustness of our conclusions.

## Conclusion

Mid-term clinical outcomes were comparable between OWHTO combined with MMPRT pull-out repair and isolated OWHTO. For patients undergoing isolated OWHTO, mechanical axis correction targeting the Fujisawa point is significantly more conducive to MMPRT healing than neutral alignment. Consider prioritizing MMPRT repair for young patients or those with high activity demands. When MMPRT repair is not performed, it is recommended to target the correction of knee alignment to the Fujisawa point.

## Data Availability

The raw data supporting the conclusions of this article will be made available by the authors, without undue reservation.
